# A meta-analysis of controlled trials of recombinant human activated protein C therapy in patients with sepsis

**DOI:** 10.1186/1471-227X-5-7

**Published:** 2005-10-14

**Authors:** Christian J Wiedermann, Nicole C Kaneider

**Affiliations:** 1 Division of Internal Medicine II, Department of Medicine, Central Hospital of Bolzano, L. Böhler Street 5, I-39100 Bolzano/Bozen (BZ), Italy; 2 Divison of General Internal Medicine, Department of Internal Medicine, Medical University of Innsbruck, Anich Street 35, A-6020 Innsbruck (Tyrol), Austria

## Abstract

**Background:**

Meta-analysis of two randomised controlled trials in severe sepsis performed with recombinant human activated protein C may provide further insight as to the therapeutic utility of targeting the clotting cascade in this syndrome.

**Methods:**

In search for relevant studies published, two randomized clinical trials were found eligible.

**Results:**

The studies, PROWESS and ADDRESS, enrolled a total of 4329 patients with risk ratio (RR) and 95% confidence interval (CI) data for effect on 28-day mortality relative to control treatment of 0.92 (0.83–1.02) suggesting that recombinant human activated protein C is not beneficial in severe sepsis. In PROWESS, 873 of 1690 patients presented with low risk, and 2315 of 2639 patients in ADDRESS as defined by APACHE II score < 25. In this low-risk stratum, no effect of recombinant human activated protein C administration on 28-day mortality was observed. This observation appears to be consistent and homogenous. Heterogeneity between the two studies, however, was seen in patients with APACHE II score ≥ 25 in whom recombinant activated protein C was effective in PROWESS (n = 817; RR 0.71, CI 0.59–0.85) whereas a tendency toward harm was present in ADDRESS (n = 324; RR 1.21, CI 0.85–1.74). Even though the overall treatment effect in this high-risk population was still in favour of treatment with recombinant activated protein C (n = 1141; RR 0.80, CI 0.68–0.94), the observed heterogeneity suggests that the efficacy of recombinant human activated protein C is not robust. Not unlikely, the adverse tendency observed could have become significant with higher statistical power would ADDRESS not have been terminated prematurely.

**Conclusion:**

This meta-analysis, therefore, raises doubts about the clinical usefulness of recombinant activated protein C in patients with severe sepsis and an APACHE II score ≥ 25 which can only be resolved by another properly designed clinical trial.

## Background

Although coagulation abnormalities may partly underlie the physiologic derangements of the sepsis syndrome, anticoagulant therapies have produced mixed results on survival in clinical studies. According to a recent meta-analysis, anticoagulants as adjuvant therapy do not appear to improve outcome in sepsis and are associated with a significant risk of bleeding complications [1]. To the extent that their treatment effect is dependent upon disease severity, the safety and efficacy of these agents may be enhanced by refinement in techniques of clinical stratification [2-4].

Recombinant human activated protein C (rhAPC) was approved for use in severely septic patients based on the results of the phase III PROWESS trial [5]. However, concerns were raised regarding rhAPC's inconsistent effects, incomplete understanding of its mechanism of action, and its safety in particular subgroups [2]. Approval was contingent among others upon agreement on post-approval clinical trials [6]. In the meanwhile, one additonal large-scale randomised controlled clinical trial has been completed, the ADDRESS study [7]. A new warning has been added to the prescribing information for rhAPC based upon exploratory analysis of the ADDRESS clinical trial database [8,9]. Because a significant portion of patients included in ADDRESS met inclusion and exclusion criteria of the PROWESS subgroup of patients for whom the current prescription labelling was given, we hypothesized that a meta-analysis of the two clinical trials of rhAPC in sepsis may provide further insight as to the therapeutic utility of targeting the clotting cascade in this syndrome with rhAPC.

## Methods

Approval of rhAPC was based on the PROWESS phase III trial [5]. Additional data on 28-day all-cause mortality and safety among adult patients with severe sepsis who were treated with rhAPC are available from the ENHANCE prospective, single-arm, multicenter clinical trial (only the US data of the trial have been published so far, [10]), and from the ADDRESS study. The single-arm ENHANCE study could enter meta-analysis only if historical controls would be used which does, however, not fulfil criteria usually applied in such type of analysis. Therefore, this analysis will focus on two studies only, i.e. PROWESS and ADRESS. Data from the PROWESS study are available from its original publication and additional material provided for the registration process of the FDA. Data from the ADDRESS have not yet been published in detail.

The primary objective of the ADDRESS trial was to demonstrate that rhAPC can reduce mortality at 28 days in adult patients with severe sepsis who have a lower risk of death, compared to placebo and conventional care. It originally planned to investigate the effectiveness of rhAPC in 11,000 adult severe sepsis patients who have a lower risk of death (APACHE score > 25; less than 2 organ failure). The trial was terminated prematurely because of futility to reach the primary study goal. The trial's data were presented at the annual meeting of the European Society of Intensive Care Medicine 2004 [7].

As approval of rhAPC is restricted to patients with severe sepsis and a high risk of death, focus of this re-analysis was on patient disease severity strata with APACHE II score < 25 and those with ≥25. Cochran-Mantel-Haenszel tests stratifying for study or for disease severity were performed together with the corresponding risk ratios (RR) and 95% confidence intervals (CI) for effect of rhAPC on 28 day mortality. For comparison pooled analyses and analyses of individual studies were performed by Fishers exact test. The homogeneity of treatment effects between strata or between studies was assessed with the Breslow-Day test.

## Results and discussion

PROWESS and ADDRESS investigated the therapeutic effects of rhAPC against placebo in patients with severe sepsis early in their disease and at a dose of 24 μg/kg/hr for a total infusion duration of 96 hours. Collectively, the studies enrolled 4329 patients (PROWESS: 1690, and ADDRESS: 2639). The RR (95% CI) for effect on 28-day mortality of rhAPC, relative to control treatment, was 0.92 (0.83–1.02) with a nominal p-value of 0.095 (Cochran-Mantel-Haenszel test). Observed heterogeneity between the two studies (Breslow-Day test, p = 0.01) suggest possible differences in treatment effects of rhAPC which may be due mainly to the higher frequency of low risk patients in ADDRESS.

In PROWESS, 48.3% (817 of 1690) of patients presented with APACHE II score ≥ 25 whereas in ADDRESS 12.3% (324 of 2639) were in this group (Tab. 1). In these patients with APACHE II score ≥ 25 inhomogeneity was observed (Breslow-Day test, p = 0.005); whereas in PROWESS, a treatment effect in favour of rhAPC was seen, a tendency toward harm was present in ADDRESS. The observed lack of homogeneity for the treatment effect with rhAPC in the two trials raises doubt regarding the robustness and the generalizability of the result. The overall effect in this patient group with higher risk remains beneficial for rhAPC. Possibly the difference between the two studies' results is due to differences in study design with a higher number of patients of this stratum in PROWESS than in ADDRESS. Another cause may lie in rhAPC's efficacy itself which has not been uniformly supported given the limitations of PROWESS [2].

**Table 1:** Meta-analysis of effects of rhAPC on 28-day mortality in patients with severe sepsis and APACHE II score of ≥25 at study entry

**Clinical Trial**	**N**	**rhAPC**	**Control**	**RR**	**95% CI**	**p-Value**
PROWESS	817	30.9	43.7	0.71	0.59 – 0.85	0.0002 ^1^
ADDRESS	324	29.7	24.5	1.21	0.85 – 1.74	0.32 ^1^
Total	1141	30.6	38.3	0.80	0.68 – 0.94	0.007 ^1,2,3^

^1 ^two-sided Fishers exact test

^2 ^Cochran-Mantel-Haenszel test, p = 0.006

^3 ^Breslow-Day test homogeneity, p = 0.005.

In PROWESS, 51.7% (873 of 1690) of patients presented with APACHE II score < 25 whereas in ADDRESS 87.7% (2315 of 2639) were in this group. In the low risk stratum no effect of rhAPC administration neither in favour nor against intervention was observed (Tab. 2). This observation appears to be consistent and homogenous (Breslow-Day test, p = 0.8).

**Table 2:** Meta-analysis of effects of rhAPC on 28-day mortality in patients with severe sepsis and APACHE II score of <25 at study entry

**Clinical Trial**	**N**	**rhAPC**	**Control**	**RR**	**95% CI**	**p-Value**
PROWESS	873	18.8	19.0	0.99	0.75–1.30	1.0 ^1^
ADDRESS	2315	16.8	16.0	1.05	0.88–1.27	0.6 ^1^
Total	3188	17.3	16.8	1.03	0.89–1.20	0.7 ^1,2,3^

^1 ^two-sided Fishers exact test

^2 ^Cochran-Mantel-Haenszel test, p = 0.007

^3 ^Breslow-Day test homogeneity, p = 0.007.

Results of this meta-analysis are interesting, firstly as they confirm in a large patient population the lack of efficacy of rhAPC in low-risk severe sepsis, and secondly suggest that beneficial treatment effects in high-risk patients may also not be so robust because patients with APACHE II score ≥ 25 did not derive benefit in ADDRESS. The adverse tendency observed could probably have become significant with higher statistical power would ADDRESS not have been terminated prematurely. Even though speculative, this might be an important aspect derived from this analysis with consequences for the clinical use or rhAPC.

The Phase II trial testing the regimen of rhAPC applied in PROWESS and ADDRESS was small with 131 patients with severe sepsis only that were studied at 4 different doses and for two different infusion periods [12]. Relevance of the findings from this study in context of the present analysis, however, is low because of patient characteristics. The predominant portion of patients had single (60%) or two-organ (33%) failure and the APACHE II score was low (mean ± SD, 17.3 ± 5.8). As this was almost exclusively a low risk patient population with a lack of data available on patients with APACHE II > 25, and due to the different dose levels which makes the study population even smaller, the Phase II study was not included in the current analysis.

Because of post-approval study obligations for the US American Food and Drug Association (FDA), Eli Lilly recently had to add a warning to the label of rhAPC (Xigris) [8]: "Among the small number of patients enrolled in PROWESS with single organ dysfunction and recent surgery (surgery within 30 days prior to study treatment), all-cause mortality was numerically higher in the Xigris group (28-day: 10/49; in-hospital: 14/48) compared to the placebo group (28-day: 8/49; in-hospital: 8/47). In a preliminary analysis of the subset of patients with single organ dysfunction and recent surgery from a separate, randomized, placebo-controlled study (ADDRESS) of septic patients at lower risk of death (APACHE II score <25 or single sepsis-induced organ failure at any APACHE II score), all-cause mortality was also higher in the Xigris group (28-day: 67/323; in-hospital: 76/325) compared to the placebo group (28-day: 44/313; in-hospital: 62/314). Patients with single organ dysfunction and recent surgery may not be at high risk of death irrespective of APACHE II score and therefore may not be among the indicated population. Xigris should be used in these patients only after careful consideration of the risks and benefits."

The European Agency for the Evaluation of Medicinal Products (EMEA) also tightened the Xigris label for the European Union including the recommendation that the product only be used in high-risk patients, mainly in situations when therapy can be started within 24 hours of the onset of organ failure and that it should only be used by experienced doctors in institutions skilled in the care of patients with severe sepsis [9].

A primary therapeutic target of rhAPC is the prevention of thrombin formation. The conflicting effects pf rhAPC in PROWESS and ADDRESS in higher risk patients raises concerns regarding original FDA and EMEA analyses suggesting that risk of death alters the effects of rhAPC in sepsis. This questions also arises in the context of other antithrombotic agents such as antithrombin III and tissue factor pathway inhibitor which can only be answered in prospectivem randomized controlled trials.

## Conclusion

This meta-analysis confirms that rhAPC is not effective in low-risk patients with severe sepsis, and it suggests that also beneficial treatment effects in high-risk patients may not be robust. It supports recent restrictive regulatory decisions in the European Union and the United States of America on Xigris and is in line with most recent developments from clinical trials, namely that Lilly has had to stop early a pediatric clinical study of Xigris after interim results showed that it was ineffective and might pose a safety risk in this population [11]. This meta-analysis raises doubts about the clinical usefulness of recombinant activated protein C in patients with severe sepsis and an APACHE II score ≥ 25 which can only be resolved by another properly designed clinical trial.

## Competing interests

CJW received fees for speaking by Ely Lilly Austria, ZLB-Behring Marburg, Germany, and Baxter Vienna, Austria.

## Authors' contributions

CJW was responsible for data acquisition, statistical analysis, and data interpretation. NCK contributed to data interpretation and was involved in drafting and writing of the manuscript.

## Pre-publication history

The pre-publication history for this paper can be accessed here:


